# Supplementary material for: Phytochemical profiling and GC-MS analysis of bioactive compounds in methanolic crude extract of Beta vulgaris (BV) root from Bangladesh
Source: PLOS Digit Health. 2025 Oct 14;4(10):e0001042. doi: 10.1371/journal.pdig.0001042 (PMC12520407; doi:10.1371/journal.pdig.0001042)
Supplement: S1 Table — (DOCX) [file pdig.0001042.s001.docx]

S1 Table. Analysis of Lipophilicity of the selected 20 molecules identified by GC-MS

| **Sl#** | **Name** | **iLOGP** | **XLOGP3** | **WLOGP** | **MLOGP** | **Silicos-IT Log P** | **Consensus Log P** |
| --- | --- | --- | --- | --- | --- | --- | --- |
| 1 | 2-Pyrrolidinone, 5-(hydroxymethyl)- | 0.85 | -1.15 | -1.12 | -0.78 | 0.37 | -0.37 |
| 2 | DL-Proline, 5-oxo-, methyl ester | 1.28 | -0.44 | -0.94 | -0.55 | 0.34 | -0.06 |
| 3 | L-Glutamine | 0.38 | -3.15 | -1.34 | -3.58 | -1.42 | -1.82 |
| 4 | Pidolic acid | 0.5 | -0.77 | -1.03 | -0.93 | -0.08 | -0.46 |
| 5 | 2-Piperidinecarboxylic acid | 1.16 | -2.31 | -0.17 | -2.21 | 0.46 | -0.61 |
| 6 | DL-Glutamic acid | 0.41 | -3.69 | -0.74 | -3.18 | -1.19 | -1.68 |
| 7 | 1,5-Pentanediol | 1.41 | -0.07 | 0.14 | 0.23 | 0.37 | 0.42 |
| 8 | Isoamyl nitrite | 0 | 1.73 | 1.73 | 0.83 | 0.96 | 1.05 |
| 9 | Cystine | 0.51 | -5.08 | -0.81 | -5.83 | -1.33 | -2.51 |
| 10 | Tetrahydro-4H-pyran-4-ol | 1.42 | -0.05 | 0.16 | -0.16 | 1.05 | 0.48 |
| 11 | Norpseudoephedrine | 1.45 | 0.83 | 0.74 | 1.26 | 1.15 | 1.09 |
| 12 | Urea, butyl- | 1.11 | 0.41 | 0.45 | 0.42 | -0.37 | 0.41 |
| 13 | Piperazine, 2-methyl- | 1.49 | -0.43 | -1.19 | -0.16 | 0.78 | 0.1 |
| 14 | Methyl tetradecanoate | 3.88 | 6.41 | 4.86 | 3.94 | 4.96 | 4.81 |
| 15 | 9-Octadecenamide, (Z)- | 4.22 | 6.99 | 5.51 | 4.16 | 5.71 | 5.32 |
| 16 | Cathine | 1.82 | 0.83 | 0.74 | 1.26 | 1.15 | 1.16 |
| 17 | Benzeneethanamine, N-methyl- | 2.75 | 2.33 | 1.87 | 2.97 | 2.64 | 2.51 |
| 18 | 3-Azabicyclo[3.2.2]nonane | 2.08 | 1.29 | 1.02 | 1.83 | 1.99 | 1.64 |
| 19 | 2-Octynoic acid | 1.95 | 2.84 | 1.73 | 1.85 | 1.45 | 1.96 |
| 20 | dl-Alanine | 0.33 | -2.96 | -0.58 | -3.06 | -1.04 | -1.46 |
