# Supplementary material for: Phytochemical profiling and GC-MS analysis of bioactive compounds in methanolic crude extract of Beta vulgaris (BV) root from Bangladesh
Source: PLOS Digit Health. 2025 Oct 14;4(10):e0001042. doi: 10.1371/journal.pdig.0001042 (PMC12520407; doi:10.1371/journal.pdig.0001042)
Supplement: S2 Table — (DOCX) [file pdig.0001042.s002.docx]

S2. Table. Analysis of Water Solubility of the selected 20 molecules identified by GC-MS

| **Sl#** | **Name** | **ESOL Log S** | **ESOL Solubility (mg/ml)** | **ESOL Solubility (mol/l)** | **ESOL Class** | **Ali Log S** | **Ali Solubility (mg/ml)** | **Ali Solubility (mol/l)** | **Ali Class** | **Silicos-IT LogSw** | **Silicos-IT Solubility (mg/ml)** | **Silicos-IT Solubility (mol/l)** | **Silicos-IT class** |
| --- | --- | --- | --- | --- | --- | --- | --- | --- | --- | --- | --- | --- | --- |
| 1 | 2-Pyrrolidinone, 5-(hydroxymethyl)- | 0.24 | 1.99E+02 | 1.72E+00 | Highly soluble | 0.61 | 4.65E+02 | 4.04E+00 | Highly soluble | -0.47 | 3.87E+01 | 3.37E-01 | Soluble |
| 2 | DL-Proline, 5-oxo-, methyl ester | -0.32 | 6.88E+01 | 4.81E-01 | Very soluble | -0.26 | 7.90E+01 | 5.52E-01 | Very soluble | -0.75 | 2.54E+01 | 1.77E-01 | Soluble |
| 3 | L-Glutamine | 1.5 | 4.65E+03 | 3.18E+01 | Highly soluble | 1.48 | 4.44E+03 | 3.04E+01 | Highly soluble | 0.68 | 6.98E+02 | 4.78E+00 | Soluble |
| 4 | Pidolic acid | -0.09 | 1.05E+02 | 8.14E-01 | Very soluble | -0.15 | 9.21E+01 | 7.14E-01 | Very soluble | -0.04 | 1.19E+02 | 9.23E-01 | Soluble |
| 5 | 2-Piperidinecarboxylic acid | 0.88 | 9.81E+02 | 7.59E+00 | Highly soluble | 1.81 | 8.34E+03 | 6.46E+01 | Highly soluble | -0.42 | 4.85E+01 | 3.76E-01 | Soluble |
| 6 | DL-Glutamic acid | 1.84 | 1.01E+04 | 6.86E+01 | Highly soluble | 2.16 | 2.15E+04 | 1.46E+02 | Highly soluble | 0.89 | 1.15E+03 | 7.83E+00 | Soluble |
| 7 | 1,5-Pentanediol | -0.18 | 6.92E+01 | 6.64E-01 | Very soluble | -0.33 | 4.89E+01 | 4.70E-01 | Very soluble | -0.68 | 2.17E+01 | 2.08E-01 | Soluble |
| 8 | Isoamyl nitrite | -1.39 | 4.75E+00 | 4.05E-02 | Very soluble | -2.16 | 8.14E-01 | 6.95E-03 | Soluble | -1.15 | 8.26E+00 | 7.05E-02 | Soluble |
| 9 | Cystine | 2.33 | 5.17E+04 | 2.15E+02 | Highly soluble | 2 | 2.39E+04 | 9.96E+01 | Highly soluble | 0.95 | 2.17E+03 | 9.02E+00 | Soluble |
| 10 | Tetrahydro-4H-pyran-4-ol | -0.44 | 3.69E+01 | 3.62E-01 | Very soluble | -0.12 | 7.78E+01 | 7.62E-01 | Very soluble | -0.13 | 7.62E+01 | 7.46E-01 | Soluble |
| 11 | Norpseudoephedrine | -1.57 | 4.05E+00 | 2.68E-02 | Very soluble | -1.38 | 6.25E+00 | 4.13E-02 | Very soluble | -1.93 | 1.78E+00 | 1.18E-02 | Soluble |
| 12 | Urea, butyl- | -0.55 | 3.24E+01 | 2.79E-01 | Very soluble | -1.13 | 8.53E+00 | 7.34E-02 | Very soluble | -1.07 | 9.91E+00 | 8.53E-02 | Soluble |
| 13 | Piperazine, 2-methyl- | -0.19 | 6.47E+01 | 6.45E-01 | Very soluble | 0.39 | 2.46E+02 | 2.45E+00 | Highly soluble | -1.17 | 6.77E+00 | 6.76E-02 | Soluble |
| 14 | Methyl tetradecanoate | -4.52 | 7.27E-03 | 3.00E-05 | Moderately soluble | -6.76 | 4.26E-05 | 1.76E-07 | Poorly soluble | -5.21 | 1.50E-03 | 6.21E-06 | Moderately soluble |
| 15 | 9-Octadecenamide, (Z)- | -5 | 2.82E-03 | 1.00E-05 | Moderately soluble | -7.71 | 5.49E-06 | 1.95E-08 | Poorly soluble | -5.61 | 6.89E-04 | 2.45E-06 | Moderately soluble |
| 16 | Cathine | -1.57 | 4.05E+00 | 2.68E-02 | Very soluble | -1.38 | 6.25E+00 | 4.13E-02 | Very soluble | -1.93 | 1.78E+00 | 1.18E-02 | Soluble |
| 17 | Benzeneethanamine, N-methyl- | -2.46 | 6.01E-01 | 3.47E-03 | Soluble | -2.04 | 1.59E+00 | 9.18E-03 | Soluble | -3.41 | 6.69E-02 | 3.86E-04 | Soluble |
| 18 | 3-Azabicyclo[3.2.2]nonane | -1.43 | 4.66E+00 | 3.72E-02 | Very soluble | -1.14 | 9.02E+00 | 7.20E-02 | Very soluble | -1.65 | 2.84E+00 | 2.26E-02 | Soluble |
| 19 | 2-Octynoic acid | -2.3 | 7.02E-01 | 5.01E-03 | Soluble | -3.28 | 7.33E-02 | 5.23E-04 | Soluble | -1.32 | 6.77E+00 | 4.83E-02 | Soluble |
| 20 | dl-Alanine | 1.54 | 3.08E+03 | 3.45E+01 | Highly soluble | 2.19 | 1.38E+04 | 1.55E+02 | Highly soluble | 0.77 | 5.23E+02 | 5.87E+00 | Soluble |
