# Supplementary material for: Phytochemical profiling and GC-MS analysis of bioactive compounds in methanolic crude extract of Beta vulgaris (BV) root from Bangladesh
Source: PLOS Digit Health. 2025 Oct 14;4(10):e0001042. doi: 10.1371/journal.pdig.0001042 (PMC12520407; doi:10.1371/journal.pdig.0001042)
Supplement: S3 Table — (DOCX) [file pdig.0001042.s003.docx]

S3 Table. Analysis of Medicinal Chemistry of the selected 20 molecules identified by GC-MS

| **Sl#** | **Name** | **PAINS #alerts** | **Brenk #alerts** | **Leadlikeness #violations** | **Synthetic Accessibility** |
| --- | --- | --- | --- | --- | --- |
| 1 | 2-Pyrrolidinone, 5-(hydroxymethyl)- | 0 | 0 | 1 | 1.5 |
| 2 | DL-Proline, 5-oxo-, methyl ester | 0 | 0 | 1 | 1.89 |
| 3 | L-Glutamine | 0 | 0 | 1 | 1.76 |
| 4 | Pidolic acid | 0 | 0 | 1 | 1.67 |
| 5 | 2-Piperidinecarboxylic acid | 0 | 0 | 1 | 1.69 |
| 6 | DL-Glutamic acid | 0 | 0 | 1 | 1.81 |
| 7 | 1,5-Pentanediol | 0 | 0 | 1 | 1.12 |
| 8 | Isoamyl nitrite | 0 | 1 | 1 | 2.24 |
| 9 | Cystine | 0 | 1 | 1 | 3.39 |
| 10 | Tetrahydro-4H-pyran-4-ol | 0 | 0 | 1 | 1.55 |
| 11 | Norpseudoephedrine | 0 | 0 | 1 | 1.62 |
| 12 | Urea, butyl- | 0 | 0 | 1 | 1 |
| 13 | Piperazine, 2-methyl- | 0 | 0 | 1 | 1.44 |
| 14 | Methyl tetradecanoate | 0 | 0 | 3 | 2.31 |
| 15 | 9-Octadecenamide, (Z)- | 0 | 1 | 2 | 2.97 |
| 16 | Cathine | 0 | 0 | 1 | 1.62 |
| 17 | Benzeneethanamine, N-methyl- | 0 | 1 | 1 | 1.54 |
| 18 | 3-Azabicyclo[3.2.2]nonane | 0 | 0 | 1 | 2.72 |
| 19 | 2-Octynoic acid | 0 | 1 | 1 | 3.73 |
| 20 | dl-Alanine | 0 | 0 | 1 | 1 |
