# Supplementary material for: Phytochemical profiling and GC-MS analysis of bioactive compounds in methanolic crude extract of Beta vulgaris (BV) root from Bangladesh
Source: PLOS Digit Health. 2025 Oct 14;4(10):e0001042. doi: 10.1371/journal.pdig.0001042 (PMC12520407; doi:10.1371/journal.pdig.0001042)
Supplement: Attachment — Submitted filename: Response to Reviewers.docx [file pdig.0001042.s004.docx]

**Response to Reviewers**

**Reviewer #1:** 
The qualitative phytochemical screening is briefly mentioned, but specific details such as reagent names, test protocols, or controls used are not included. These should be described or referenced to ensure reproducibility.

The GC-MS method lacks critical information such as instrumentation parameters, retention times, column type, and identification approach (e.g., comparison with NIST library).

**Response:** Dear reviewer, thank you very much for reviewing and pointing out the need for more detailed information on the qualitative phytochemical screening. We have revised the manuscript to provide specific details, including the names of reagents used, test protocols for each phytochemical class, and the positive and negative controls.

| **Phytochemicals** | **Test Method** | **Protocol** | **Standard control** | **Reagents and Manufacturer** |
| --- | --- | --- | --- | --- |
| Alkaloids | Wagner’s test | 0.5 ml of extract was dissolved in 1 ml of 1% (v/v) HCl, later, 3 drops of Wagner’s reagent were added to the solution. | Caffeine | Caffeine and Wagner’s reagent, Sigma Aldrich (USA) |
|  | Dragendorff test | 1 ml of Dragendorff’s reagent was added to 2 ml of plant extract | Caffeine | Dragendorff’s reagent, Merck (Germany) |
| Flavonoids | Alkaline reagent assay | 2-3 drops of sodium hydroxide (NaOH) were added to 2 mL of plant extract (1mg/mL); later, 5 mL dilute HCl was added. | Quercetin | Quercetin and NaOH, Merck (Germany) |
| Polyphenols | Folin-Ciocalteu test | 0.5 mL of a different solvent extract (1 mg/ml) was combined with 2 mL of Folin & Ciocalteu′s phenol reagent (10X diluted); after 5 minutes 2.5 mL of 7.5% of Na₂CO₃. | Gallic acid | Folin & Ciocalteu′s phenol reagent, Merck (Germany) |
| Tannins | Lead acetate test | Three drops of 1% (w/w) ferric chloride (FeCl₃) to 1 mL of plant extract. | Gallic acid | FeCl₃, Merck (Germany) |
| Steroids | **Liebermann-Burchard's test** | 2 ml of acetic anhydride and 2 mL of concentrated H_2_SO_4_ were added to 5 ml of plant extract | - | Acetic anhydride, Merck (Germany) |
| Terpenoids | Salkowski Test | 2 ml of chloroform with 1-2 drops of concentrated H_2_SO_4_ was added to 5 mL of plant extract. | Citral | Chloroform, Merck (Germany) |
| Carbohydrates | Molisch’s Test | 2–3 drops of Molisch’s reagent were added to 2 mL of beetroot extract, followed by the gentle addition of 1 mL of concentrated H₂SO₄. | Glucose | Molisch’s reagent, Merck (Germany) |

Please have a look in the materials and methods section of the revised manuscript (line no 99-109 and 138-169)

We have revised the manuscript and included comprehensive information on the instrumentation and analytical parameters for GC-MS analysis.

| **Instruments** | **Set parameters** |
| --- | --- |
| Column type | Rtx-5MS fused silica capillary column (30 m × 0.25 mm i.d., 0.25 µm film thickness) |
| Carrier gas flow rate | Carrier gas (Helium) flow rate: 1.1 ml/min |
| Oven temperature program | 90°C at 0 min, increased to 200°C for 2 min (3°C/min), then 280°C for 2 min (15°C/min) |
| Retention times | 50 minutes |
| Identification approach | Mode: auto scan mode; Library: NIST‐MS Library (Ver. 3.4.5) |

For more details, please have a look in the materials and methods section of the revised manuscript (line no 203-217)

**Reviewer #2:**

The manuscript is of interest because it explores bioactive compounds in a Beta vulgaris (beetroot) extract from Bangladesh and combines conventional phytochemical tests, gas chromatography–mass spectrometry (GC-MS) analysis, and in silico evaluations of pharmacokinetic properties. This multidisciplinary approach may help identify lead compounds for further drug development studies. The author also points out the lack of findings in phytochemical profiling of the beetroot grown in Bangladesh. The findings from this also add to the abundance of data in profiling beta vulgaris for phenotyping and comparisons with other origins from other geographical areas.

Strengths

1. Comprehensive Methodology Integration: The study integrates a multifaceted approach to not only profiling metabolite classes and identifying major constituents, but also performs drug-likeliness analysis, strengthening the findings.

2. Pharmacokinetic Modeling Transparency: The manuscript explicitly describes estimation formulas for key parameters (logBB, plasma protein binding (%PPB), half-life). For example, logBB=log(cerebral/plasma concentration) clarifies the physiological relevance of BBB permeability predictions.

3. GC-MS and In Silico Synergy: The combination of experimental chromatographic data with computational tools demonstrates practical applications in early drug discovery pipelines. Similar methodologies can be implemented for other use cases, especially given the highly reproducible description of the approach taken by the study team.

Potential Limitations

1. Ambiguous Formula Derivations: Equations like %PPB=dlogP+eMW+fHBA+g reference undefined coefficients (d,e,f,g). I presume they originate from the SwissADME models, and hence to the author, the models might be a black box and would limit access to coefficients/ weights. Additionally, Clarification on whether these models are generalized or empirically tailored to beetroot compounds. Include citations for original model derivations if applicable, might increase generalizability of the method.

2. Availability of data: The manuscript indicates “All data underlying the findings are fully available without restriction.” Please provide a precise statement regarding where and how the datasets can be accessed (e.g., repository name, accession numbers, DOIs) so that interested readers and reviewers can verify the results.

3. Software References: Chemaxon’s MarvinSketch and SwissADME tools are cited adequately (with URLs for Chemaxon), but versions or default parameters used lack specificity.

4. Comparison with other geographical areas: Expand discussion on elucidating the difference found by your identified compounds compare with those reported in other geographical or botanical studies.

Conclusion

This manuscript offers a robust computational framework integrating structural analysis with pharmacokinetic predictions. With revisions addressing these gaps, the study contributes a valuable methodology resource for early-stage drug discovery pipelines.

**Response:** We sincerely thank the reviewer for addressing the potential strength of our manuscript, citing the value of our integrated experimental and computational approach.

We also thank the reviewer for pointing out potential limitations to improve our manuscript. To avoid ambiguity, we omitted these equations in the revised manuscript. We also rewrote paragraphs of the materials and methods section related to the visualization of chemical structures, estimation of physicochemical properties of the identified molecules by GC-MS

Please have a look in the revised manuscript (line no 218-245)

We mentioned *“All data are in the manuscript and/or supporting information files”* during the submission of revised manuscript.

We have updated the manuscript to include the exact versions of the software: Chemaxon’s MarvinSketch version (version 24.3.0) and SwissADME (online version). Additionally, we have clarified the default parameters used for calculations, including pH, temperature, and other relevant settings, to ensure reproducibility of our analyses.

Please have a look in the revised manuscript (line no 218-245)

We have expanded the Discussion section to compare the phytochemical profile identified in beetroot cultivated in Bangladesh with those reported in other geographical regions. This includes highlighting similarities and differences in the presence and relative abundance of key bioactive compounds, as well as possible influences of environmental factors, soil composition, and cultivation practices. Relevant literature references from studies conducted in different countries have been added to contextualize and generalize our findings.

Please have a look in the revised manuscript (line no 497-513)

**References:**

7.	Fiadorwu J, Subedi K, Todd D, Basti MM. Multipronged Approach to Profiling Metabolites in Beta vulgaris L. Dried Pulp Extracts Using Chromatography, NMR and Other Spectroscopy Methods. Foods. 2023;12(18). Epub 2023/09/28. doi: 10.3390/foods12183510. PubMed PMID: 37761219; PubMed Central PMCID: PMCPMC10528680.

57.	Üst Ö, Yalçin E, Çavuşoğlu K, Özkan B. LC-MS/MS, GC-MS and molecular docking analysis for phytochemical fingerprint and bioactivity of Beta vulgaris L. Sci Rep. 2024;14(1):7491. Epub 2024/03/30. doi: 10.1038/s41598-024-58338-7. PubMed PMID: 38553576; PubMed Central PMCID: PMCPMC10980731.

58.	Kusznierewicz B, Mróz M, Koss-Mikołajczyk I, Namieśnik J. Comparative evaluation of different methods for determining phytochemicals and antioxidant activity in products containing betalains - Verification of beetroot samples. Food Chem. 2021;362:130132. Epub 2021/06/04. doi: 10.1016/j.foodchem.2021.130132. PubMed PMID: 34082297.

59.	Youssef NH, Qari SH, Behiry SI, Dessoky ES, El-Hallous EI, Elshaer MM, et al. Antimycotoxigenic Activity of Beetroot Extracts against Alternaria alternata Mycotoxins on Potato Crop. Applied Sciences [Internet]. 2021; 11(9).
